# Exogenous bovine somatotropin and mist-fan cooling synergistically promote the intramammary glucose transport for lactose synthesis in crossbred Holstein cows in the tropics

**DOI:** 10.14202/vetworld.2021.1247-1257

**Published:** 2021-05-21

**Authors:** Narongsak Chaiyabutr, Siravit Sitprija, Somchai Chanpongsang, Sumpun Thammacharoen

**Affiliations:** 1 Department of Physiology, Faculty of Veterinary Science, Chulalongkorn University, Bangkok 10330, Thailand; 2 Academic of Science, Royal Society of Thailand, Bangkok, Thailand; Queen Saovabha Memorial Institute, The Thai Red Cross Society, Bangkok 10330, Thailand; 3 Department of Biology, Faculty of Science, Mahidol University, Bangkok, Thailand; 4 Department of Animal Husbandry, Faculty of Veterinary Science, Chulalongkorn University, Bangkok 10330, Thailand

**Keywords:** crossbred Holstein cattle, lactose, milk glucose, milk glucose-6-phosphate, mist-fans cooling, recombinant bovine somatotropin

## Abstract

**Background and Aim:**

Milk synthesis by the mammary gland is negatively influenced in part by high ambient temperature (AT). This study aimed to clarify the pathway of intramammary glucose utilization involved in mediating lactose synthesis during treatment with somatotropin under housing with misters and fans.

**Materials and Methods:**

A single subcutaneous injection of 500 mg of recombinant bovine somatotropin (rbST) was administered 3 times once every 14 days to 87.5% crossbred Holstein cattle in early-/mid-/late lactation, under housing in a normal shaded barn and in a shaded barn with a mist-fan cooling system.

**Results:**

The milk yields of the cooled cows tended to increase compared with those of uncooled cows and exhibited more potentiated effects in response to rbST treatment, coinciding with increases in mammary plasma flow and glucose uptake, but not in the mammary extraction of glucose. Treatment with rbST in the cooled cows resulted in a greater increase in the milk glucose concentration and a greater decrease in the milk glucose-6-phosphate concentration at all stages of lactation.

**Conclusion:**

rbST treatment exerted its galactopoietic action more by local intramammary factors than by other extramammary factors at a low AT and the synergistic effect between rbST treatment and low AT increased the availability of intramammary glucose transport in activating the process of lactose synthesis.

## Introduction

The low milk yield and shorter persistency of lactation in either pure exotic or crossbred dairy cattle remain major problems in the dairy industry in the tropics. The mechanisms responsible for limiting the milk yield in crossbred dairy cattle under tropical conditions are unclear; although *Bos taurus*×*Bos indicus* crossbred dairy cattle have been exploited as an efficient option for mixing the adaptability of tropical cattle with the high milking potential of exotic breeds to maximize milk production. The previous studies on 87.5% crossbred Holstein (Holstein-Friesian [HF]) dairy cows at a tropical temperature indicated that the downregulation of milk secretion at mid-to late lactation in the tropics could be explained by metabolic adaptations [1]. These adaptations include a reduction in the level of plasma bovine somatotropin (bST) accompanying reductions in mammary blood flow (MBF) and nutrient uptake by the mammary gland. However, these changes did not provide a conclusive explanation of the specific mechanism involved.

Although exogenous growth hormone (GH) is a widely used model for studying galactopoiesis for milk production in dairy cows, its mechanisms of action in regulating milk synthesis in the mammary gland of crossbred dairy cows in the tropics is unclear. Studies in 87.5% HF cows kept under a tropical temperature revealed that prolonged recombinant bST (rbST) treatment exerted its galactopoietic action through an increase in milk yield, at least in the early stage of lactation, but the stimulant effect on milk yield was reduced in the late lactation stage despite a high MBF level [2]. The regulation of biosynthetic capacity within the mammary gland during prolonged rbST treatment demonstrated that the proportion of utilized glucose in the mammary gland that was metabolized for lactose synthesis was reduced, causing a decline in milk yield with advanced lactation [3]. These studies did not establish a relationship between the mechanism of action of bST and the effects of a high environmental temperature, including which factors were the cause and which factors were the effects of such change, although a high environmental temperature would be another factor affecting milk production in dairy cows in the tropics [4]. Several investigations have reported inconsistent observations about the action of bST treatment in dairy cows and goats kept at a high environmental temperature. It was reported that dairy cows and goats treated with bST under short-term exposure to hot environmental conditions increased their milk production and the hot environmental conditions did not reduce these bST effects [2,5]. Other investigators reported that a high ambient heat load reduced the response to bST treatment and conferred low milk yields [6], while heat-stressed cows had an elevated rectal temperature (RT) and respiratory rate (RR), which was exacerbated in cows receiving bST treatment [7]. However, these lines of evidence were not definitive since another study in dairy cows showed that the reduction in milk yield during heat stress was modulated by a negative regulatory feedback system present in milk [4]. Against this background, the question arises of whether low milk production in crossbred dairy cows is due to the effect of a lower bST level *per se*, or from the combination of the effect of a high environmental temperature modulating a negative regulatory feedback system, where these combined effects on the mammary gland function remain obscure. Many investigations and strategies can be used to alleviate the impact of a hot environment with modification of the cooling systems to increase the quality and efficiency of milk production in dairy cows [8].. Along with these aims, various cooling techniques have been reviewed [9]. Considering the obtained data, it was previously hypothesized by our group that a lower environmental temperature that modulates bST action would upregulate milk production in crossbred dairy cows in the tropics. Indeed, the effects of mist-fan (MF) cooling combined with recombinant rbST treatment on milk production in crossbred HF cows were shown to involve changes in both intra- and extra-mammary parameters, where 87.5% HF cows housed under MF cooling showed a reduction in the negative effect of high temperatures on digestive function through an increase in the digesta passage rate, resulting in increased feed intake in the supply of nutrients to the mammary gland [10]. The increased dry matter intake (DMI) in response to the combined MF system and rbST treatment was attributed to increased rumen fermentation with consequential increases in the levels of available volatile fatty acids, NH_3_N, and microbial proteins [10]. The rbST-treated cows housed in an MF barn showed a high level of water absorption through the ruminal wall, which would in part account for the increased total body water level during rbST treatment [11]. This would then affect the distribution of nutrients to the mammary gland and thermoregulatory mechanisms. In addition, an interaction between the metabolic hormones circulating during rbST treatment in both cooled and uncooled cows was reported [12], especially a marked increase in plasma insulin-like growth factor I (IGF-I). IGF-1 is an important endocrine mediator of the effect of rbST by increasing the MBF, which enhances the availability of substrates to the mammary gland for milk synthesis. In addition, a low plasma leptin level occurred under the cooling management, which coincided with the lipolytic effect of rbST, and subsequently increased the DMI and supply of nutrients to the mammary gland in both MF-cooled and uncooled cows. In both cooled and uncooled cows as lactation advanced to the late stage, whether treatment with rbST was applied or not, the glucose uptake by the mammary gland was metabolized less for lactose synthesis but metabolized more through the Embden–Meyerhof pathway and the tricarboxylic acid cycle, while the glucose turnover rates and plasma glucose concentrations were not significantly different [11]. The administration of exogenous rbST in lactating HF cows, both cooled and uncooled, increased the milk yield without adverse effects on hematological and biochemical parameters [13]. Together, these findings suggest that the effects of rbST treatment on milk production in cows housed in MF barns (hereafter MF cows) are due in part to changes in the extramammary functionality in providing substrates, either directly or indirectly, to the mammary gland, which results in increased milk production. However, the effects of cooling in cows alone or in combination with rbST treatment on the local changes in their capacity to biosynthesize substrates relevant to milk production within the mammary gland have not been clarified.

Milk secretion is primarily driven by lactose secretion, which plays a primary role in the regulation of milk volume within the mammary epithelial cells [14]. An increase in the yield of milk lactose by the galactopoietic effect of GH without influencing the concentration of lactose in lactating ruminants has been shown [15]. However, no definitive conclusions have been drawn on the mechanisms acting within the mammary gland to increase the rate of milk secretion in MF cows cooling in combination with rbST treatment. Lactose synthesis is closely linked to glucose utilization from free glucose and uridine diphosphate (UDP)-galactose by lactose synthase in the lumen of the Golgi apparatus in mammary epithelial cells. Thus, glucose is the principal precursor for the synthesis of lactose and participates in determining the volume of milk produced. The intracellular glucose level in mammary epithelial cells is dependent on the quantity of glucose absorbed from the blood, since mammary epithelial cells do not synthesize glucose due to their lack of glucose-6-phosphatase [16]. An understanding of the changes in the availability of glucose within the mammary gland is needed because the metabolism of intracellular glucose in mammary epithelial cells has many roles, including the conversion to glucose-6-phosphate (G6P), an intermediate compound for the generation of UDP-galactose during lactose synthesis, and the first step in both glycolysis and the pentose phosphate pathway. Free glucose and G6P in secreted milk are assumed to represent the intracellular level at the time of secretion from mammary epithelial cells, which are related to the difference between the rate of glucose transport into the cell and the rate of its intracellular utilization [14]. Measurement of the concentrations of milk metabolites reflecting the concentrations of intracellular components has been shown to be a useful, non-invasive method of following changes in mammary metabolism without using tissue samples [17]. Therefore, the concentrations of either glucose or G6P in milk could reflect the intracellular glucose transport and metabolism, including the physiological status of mammary function. However, whether intracellular glucose and G6P in mammary cells modulate the pattern of lactose synthesized *de novo* and milk secretion in response to external factors (either the environmental temperature or treatment with rbST) has still not been clarified in crossbred dairy cows.

Accordingly, the present study was designed to test the hypothesis that rbST treatment and exposure to a lower environmental temperature through MF cooling enhance the availability of intramammary glucose transport for lactose synthesis in a synergistic process for milk secretion. To achieve this, we measured the concentrations of glucose and G6P in milk as biomarkers to evaluate the pathway of intramammary glucose transport in crossbred 87.5% HF cattle treated with rbST and housed in MF barns (MF cows) in comparison with those housed in uncooled barns (hereafter NS cows). We also investigated whether synergism between the effect of rbST and a low ambient temperature (AT) (from MF cooling) increased the availability of intramammary glucose transport to activate lactose synthesis in different stages of lactation.

## Materials and Methods

### Ethical approval

All animal experiments were approved by the Experimental Animal Ethics Committee of the Faculty of Veterinary Science, Chulalongkorn University (Protocol No 0831029). Moreover, the study was conducted in compliance with the guidelines of the National Research Council of Thailand for the care and the use of experimental animals. Animal facility staff, as well as research staff were trained in the correct and humane handling of dairy cattle before the start of any procedure.

### Study period and location

All the dairy cows were raised at dairy farm at Veterinary Student Training Center, Chulalongkorn University, Nakhon Pathom Province (Latitude 13º49’10.56 N, Longitude 100º2’39.37 E and at 20 meters above sea level), which is situated approximately 55 km. West of Bangkok. The experiment was performed from March 2018 to August 2019, when the environmental condition was high AT[18]. Annual average of meteorological data is AT=29-35°C, Relative Humidity (RH)=72-80%.

### Animals, housing, and management

The study presented here is part of a more extensive study in the research package of the research grant (“Physiological responses of lactating crossbred Holstein cattle to high AT and control mechanisms to reduce its effect on milk production”).

The pattern of experimental protocols of this study followed those previously described [12]. In brief, ten primiparous, non-pregnant cattle, containing 87.5% HF genes, were assigned into two groups of five cows each based on body weight (358±32.5 kg). Cows at an average of 60 days postpartum at the start of the trial were used for the experiment. Cows in the first group were housed in an open-sided barn (8 m long×7 m wide×3.5 m high) with a tiled roof in a normal shaded house as the uncooled NS cows. Cows in the second group were housed similarly, except for the provision of misters and fans (MF) as cooled MF cows. The open space cooling system consisted of two sets of MF, in which each consisted of a 65-cm-diameter blade fan circulating 81 m^3^/min of air, with oscillation coverage of 180º, while water was discharged from four spray heads at 7.5 L/h with a mist droplet size of 0.01 mm. Cooled cows were exposed to MF for 45 min at 15-min intervals using an automatic electric time clock controller from 06:00 h to 18:00 h and were exposed to MF for 15 min at 45-min intervals from 18:00 h to 06:00 h.

The AT was recorded using a dry bulb thermometer. The RH was read depending on the wet and dry bulb temperatures at the cooled and uncooled barns. The AT and RH were measured weekly at 14:00 h on the specified day. Average values were considered to be the mean of all measurements taken for each date. Temperature humidity index (THI) was calculated according to the National Research Council’s equation [10], as follows: THI=0.72 (Tdb+Twb)+40.6, where Tdb is the dry bulb temperature (°C) and Twb is the wet-bulb temperature (°C).

The feeding regimens of dairy cattle were similar between the two groups. The physical composition of the total mixed ratio (TMR) diet was (by % weight) as follows: Pineapple waste (50%), soybean meal (23%), rice bran (3.0%), cottonseed (20%), limestone (1.4%), di-calcium phosphate (1.4%), sodium bicarbonate (0.3%), potassium chloride (0.1%), and mineral and vitamin premix (0.8%). The chemical composition for dry matter (DM) was 40%. Other chemical compositions (% DM) were as follows: 13% for ash, 87% for organic matter, 19% for crude protein, 20% for acid detergent fiber, and 30% for neutral detergent fiber. The TMR diet was fed twice a day *ad libitum* at the same ratio throughout the experiments in both groups.

Cows were milked twice a day using a milking machine and milk yields were recorded at each milking. Each day, the food was given in equal portions at about 06:00 h and 17:00 h when animals were milked. The RT and RR were measured weekly at 14:00-15.00 h on the specified day, the former by electronic thermometers and the latter by observing flank movements.

### Experimental design

The experiment in each group was divided into three stages of early (2 months postpartum), mid- (4 months postpartum), and late lactation (6 months postpartum). The pre-treatment was conducted on the 1^st^ day of each stage of lactation. At the end of the pre-treatment period, within the same day, each cow was injected with the first dose of 500 mg of rbST (POSILAC; Monsanto, USA) with two consecutive 500 mg doses every 2 weeks. Thereafter, within 2-5 days after the third rbST injection, the experiments for the treatment period were conducted. Therefore, the protocol of the studies involved the period of pre-treatment, three doses of rbST injections, and the period of treatment. This protocol was performed during the first 30 days and the same procedures were followed for each stage of lactation. During the last 30 days of each stage of lactation, no experiments were conducted to allow the milk yield to return to the control level. Thus, comparative studies in the short experimental periods (30 days between pre-treatment and treatment) were performed in both groups of cows (MF and NS cows) in each stage of lactation, which means that the milking day should not have influenced the milk yield.

### Determination of the mammary plasma flow (MPF)

The MPF was measured by the dye dilution technique using the dye T-1824 (Evans blue) during 10.00-11.00 h on the specified day, as previously described [2]. A venous blood sample was collected from the milk vein through a catheter, while an arterial blood sample was collected from the coccygeal vessel by venipuncture with a 21-gauge needle. Blood samples in heparinized tubes were kept in crushed ice until centrifugation to isolate plasma samples, which were then used for determination of the dye dilution and plasma glucose concentrations using glucose oxidase.

### Collection of milk samples and estimation of milk metabolites

On each specified day, a milk sample was collected at the evening milking from each animal, and followed by the measurement of the MBF and mammary glucose uptake on the day of each period of study. The milk sample was then kept at 4°C until the next day, when it was defatted, deproteinized, and then analyzed for the concentration of metabolites. Briefly, the milk was centrifuged at 9391 g for 15 min at 4°C. The aqueous phase below the solidified fat layer was removed. The supernatants were used to determine the milk glucose concentration using glucose oxidase, while G6P was determined by colorimetric tests using a G6P assay kit (Sigma-Aldrich, USA).

The milk citrate and lactose concentrations were determined colorimetrically as previously described [11]. The concentration of electrolytes in milk was estimated for sodium (Na^+^) and potassium (K^+^) using a flame photometer, while chloride (Cl^−^) was measured using a chloridometer (Chloride analyzer 925; Ciba Corning Inc., USA). The osmolality of milk was determined using an osmometer (Osmometer 3D3; Advance Instruments Inc., USA). Mammary uptake of glucose was estimated from the MPF and arteriovenous (A-V) concentration difference of glucose in plasma. Uptake of glucose by the mammary gland, expressed as mol/min, was calculated from the equation: Mammary uptake = MPF × (PA − PV). Here, MPF is in mL/min, PA is the concentration of glucose in the coccygeal arterial plasma (mol/mL), and PV is the concentration of glucose in the plasma from the milk vein (mol/mL).

### Statistical analysis

Statistical analyses were performed using the General Linear Model procedures of the SPSS statistical software package (SPSS for Windows, V14.0; SPSS Inc., Chicago, IL, USA). The model used for each analysis was as follows:

Y_ijk_ = µ+A_l_+H_i_+A(H)_il_+B_j_+(HB)_ij_+A(HB)_ijl_ +Cov_k_+e_ijkl_

where Y_ijk_ is the observation, µ is the overall mean, A_l_ is the animal effect, H_i_ is the house effect as the main plot (*i*=NS, MFC), A(H)_il_ is the main plot error (animal *l* in house *i*), B_j_ is the treatment effect (rbST) as a split plot (*j* is with and without rbST supplementation), (HB)_ij_ is the interaction effect between treatment and house, A(HB)_ijl_ is the split plot error (animal *l* in house *i* and treatment *j*), Cov_k_ is the covariate effect, and e_ijk_ is the residual error.

In addition, Student’s unpaired t-test was used to compare the overall effects of rbST treatment on the concentration or concentration ratio of milk glucose, milk G6P, and lactose of pooled data obtained from both MF and NS cows in all stages of lactation. Likewise, overall comparative effects of cooling on percent changes of the milk glucose and milk G6P concentrations during rbST treatment in both MF and NS cows at all stages of lactation were determined. p<0.05 was considered to indicate a statistically significant difference and a trend was identified at a p<0.10.

## Results

### Changes in environmental (AT, RH, and THI) and physiological (RT and RR) parameters

**Figure-1:**
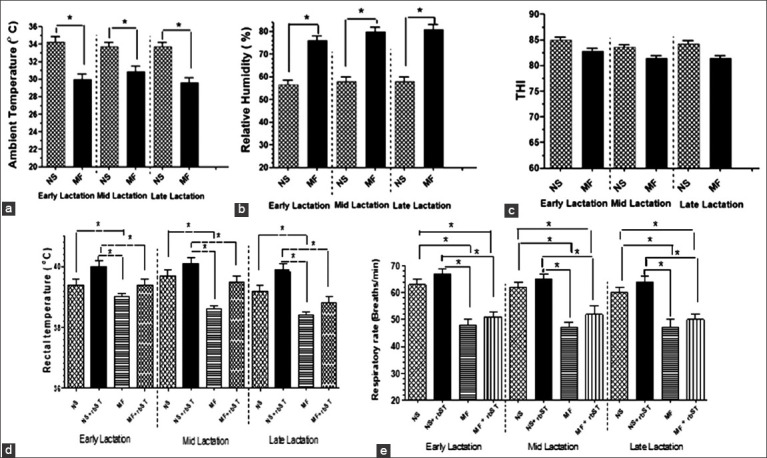
Comparative measurements of (a) the ambient temperature, (b) relative humidity and (c) temperature humidity index (THI) at different periods of lactation at normal non-cooled shaded house (natural service [NS]) and cooled housed provision of misters and fans (MF). The ambient temperature, relative humidity, and THI were measured at 14.00 h. Rectal temperature (D), and respiratory rate (E) of recombinant bovine somatotropin-treated MF- and NS-cows at different stages of lactation. Data are shown as the mean±SD. Comparison of significant differences of mean values between parameters in different stages of lactation, at *p<0.05 using Student’s unpaired t-test.

The environmental parameters (AT, RH, and THI) and physiological parameters (RR and RT) are summarized in Figure-1. The ATs in the NS barn (average 34°C) were significantly higher (p<0.05) than those in the MF barn (average 30°C), but the RH of the MF barn (average 78%) was significantly higher (p<0.05) than that of the NS barn (average 57%) throughout the study period (i.e., at all stages of the MF and NS cows’ lactation). However, the THI varied, it was not significantly different between the MF barn and the NS barn (averages of 84 and 82, respectively). The RR and RT of the MF cows were lower than those of the NS cows treated with rbST at all stages of lactation. During treatment with rbST, the RR and RT were significantly higher (p<0.05) than those in the pre-treatment periods in both MF and NS cows in all stages of lactation.

### Levels of MPF, plasma glucose concentration, mammary glucose uptake, and milk yield

During rbST treatment, the MPF was significantly higher (p<0.05) than the pre-treatment values in both MF and NS cows at each stage of lactation (Table-1). The mean values for the plasma glucose concentration in both the MF and NS cows showed no significant changes during rbST treatment compared with those in the pre-treatment period. The uptake of glucose across the udder increased in each stage of lactation in both MF and NS cows treated with rbST, and these effects were significant in mid-lactation (p<0.05), while showing a tendency for significance at the late stage of lactation (p<0.1). The milk yields of both MF and NS cows treated with rbST were significantly higher than those in the pre-treatment at each stage of lactation. Because lactose secretion is the function of lactose concentration and milk yield, the secretion of lactose from both MF and NS cows apparently increased during rbST treatment, while no alterations in the milk lactose concentrations were apparent.

**Table-1:** Milk yield, MPF, arterial plasma glucose concentration, A-V difference, and mammary glucose uptake in MF- and NS-cows with or without supplemental rbST treatmentat different stages of lactation in crossbred HF dairy cattle.

Parameter	Lactation	NS		MF		SEM	^1^Effect		
		
		Pre	rbST	Pre	rbST		MF	rbST	MF x rbST
Milk yield (kg/d)	Early	10.97	12.64	12.15	13.04	0.492	0.458	0.044	0.385
	Mid	9.11	10.74	11.37	13.47	0.573	0.285	0.035	0.306
	Late	8.05	9.64	9.24	12.24	0.537	0.368	0.009	0.412
MPF (mL/min)	Early	3452	3822	3761	4771	179	0.665	0.007	0.064
	Mid	2388	3536	3370	4101	151	0.834	0.033	0.275
	Late	2494	3816	3152	3533	199	0.776	0.006	0.143
Plasma flow/ Milk yield (L/kg)	Early	453	435	446	526	55	0.939	0.686	0.324
	Mid	377	474	427	438	76	0.658	0.910	0.385
	Late	446	570	496	415	78	0.654	0.527	0.553
Glucose: Arterial plasma (µmol/mL)	Early	3.40	3.50	3.69	3.59	0.09	0.102	0.788	0.776
	Mid	3.26	3.44	3.37	3.40	0.10	0.876	0.745	0.215
	Late	3.63	3.64	3.78	3.64	0.08	0.889	0.342	0.557
A-V difference (µmol/mL)	Early	0.61	0.66	0.64	0.56	0.09	0.820	0.296	0.470
	Mid	0.66	0.69	0.59	0.65	0.06	0.909	0.650	0.729
	Late	0.87	0.79	0.78	0.80	0.04	0.906	0.195	0.125
Mammary uptake (µmol/min)	Early	2124	2550	2412	2662	256	0.678	0.157	0.635
	Mid	1588	2446	1981	2651	347	0.553	0.045	0.882
	Late	2204	3034	2506	2838	297	0.490	0.053	0.363

SEM=Standard error of the mean.

1 p-values for the effects; MF=Mist-fan cooling effect, of rbST; rbST=treatment period of rbST. Significance effect; p<0.05 and trends are declared at 0.05<p<0.10.

### Concentrations of lactose, glucose, G6P, citrate, Na^+^, K^+^, and Cl^−^, and the osmolality of the milk

**Figure-2:**
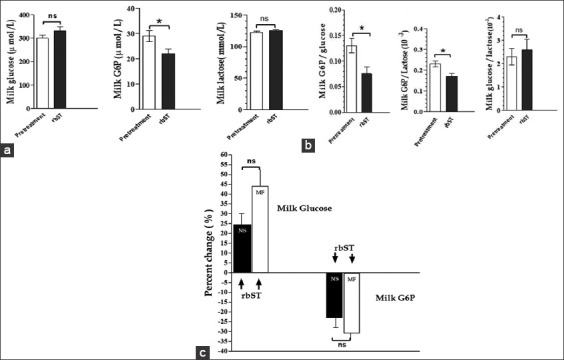
Changes in the (a) concentrations and (b) concentration ratio of lactose, glucose, and G6P in mammary secretions, and (c) comparable percent changes in the concentrations of glucose and G6P in mammary secretions in recombinant bovine somatotropin-treated misters and fans- or natural service-cows at all stages of lactation. Data are shown as the mean±SD, (p-values by Student’s unpaired t-test where *p<0.05, ns=Not significant).

No changes in the milk lactose concentrations were apparent throughout the experimental periods (Table-2). However, both MF and NS cows treated with rbST showed an insignificant increase in milk glucose concentration in early and mid-lactation, while the magnitude of the increase in milk glucose concentration was lower during rbST treatment in the late stage of lactation compared with that in the early stage of lactation in the MF and NS cows. The milk G6P concentrations significantly decreased (p<0.05) during the rbST treatment in the MF and NS cows through all stages of lactation (Figure-2a). Variations were found between the ranges of concentrations of glucose and G6P in the milk.

**Table-2:** Milk metabolites and electrolytes during rbST treatment in MF- and NS-cows at different stages of lactation.

Parameter	Lactation	NS		MF		SEM	^1^Effect		
		
		Pre	rbST	Pre	rbST		MF	rbST	MF x rbST
Lactose (mmol/L)	Early	136.1	141.7	137.8	138.1	2.17	0.845	0.435	0.224
	Mid	138.9	141.4	138.9	141.9	2.54	0.837	0.765	0.365
	Late	137.2	139.4	140.8	134.7	2.67	0.634	0.408	0.453
Free glucose (µmol/L)	Early	288.9	333.3	222.2	397.8	77.9	0.989	0.196	0.425
	Mid	246.7	340.0	355.6	426.7	79.4	0.252	0.331	0.892
	Late	280.0	288.9	211.1	288.9	77.0	0.448	0.435	0.305
G6P (µmol/L)	Early	61.8	43.9	32.5	22.6	9.05	0.393	0.378	0.539
	Mid	53.8	49.2	25.2	18.4	8.53	0.276	0.117	0.551
	Late	38.8	27.2	28.9	18.8	5.92	0.540	0.157	0.337
Glucose**/**lactose (m/m)10^3^	Early	2.14	2.35	2.18	2.98	0.31	0.951	0.494	0.172
	Mid	2.11	2.54	2.67	3.20	0.90	0.321	0.515	0.318
	Late	2.84	2.22	1.68	2.19	0.34	0.284	0.975	0.937
G6P**/**lactose (m/m )10^3^	Early	0.48	0.31	0.45	0.33	0.09	0.820	0.296	0.470
	Mid	0.39	0.35	0.40	0.36	0.06	0.909	0.650	0.729
	Late	0.29	0.21	0.31	0.21	0.04	0.906	0.195	0.125
G6P/glucose m/m)	Early	0.24	0.21	0.17	0.07	0.04	0.582	0.220	0.329
	Mid	0.22	0.14	0.12	0.06	0.02	0.146	0.070	0.299
	Late	0.10	0.11	0.20	0.15	0.03	0.195	0.486	0.572
Citrate (mmol/L)	Early	4.25	4.56	4.24	4.86	0.25	0.301	0.044	0.298
	Mid	4.71	4.74	5.69	5.79	0.16	0.161	0.581	0.647
	Late	4.75	4.18	5.23	4.41	0.12	0.223	0.115	0.398
Na^+^ (mmol/L)	Early	29.80	31.20	27.20	27.60	1.31	0.250	0.449	0.670
	Mid	28.80	29.20	27.80	28.00	0.79	0.651	0.714	0.902
	Late	32.60	32.20	29.00	32.00	2.05	0.700	0.544	0.432
K^+^ (mmol/L)	Early	38.70	36.88	36.80	36.04	1.27	0.434	0.341	0.688
	Mid	37.06	36.16	36.32	35.08	0.90	0.582	0.267	0.854
	Late	35.16	34.74	33.84	34.50	1.07	0.609	0.914	0.628
Cl^-^ (mmol/L)	Early	31.00	33.00	36.40	35.80	0.78	0.277	0.398	0.136
	Mid	33.20	32.80	29.80	27.60	0.78	0.255	0.132	0.279
	Late	33.40	32.60	33.80	41.60	3.75	0.541	0.378	0.284
Osmolality (mOsm/kg)	Early	277.6	277.8	279.6	279.0	3.37	0.710	0.754	0.908
	Mid	276.6	272.6	279.8	277.2	1.66	0.289	0.081	0.684
	Late	269.8	275.0	302.2	283.4	9.60	0.265	0.499	0.246

SEM=Standard error of the mean.

1 p-values for the effects; MF=Mist-fan cooling effect, rbST=rbST effect, MF×rbST=Interaction effect of MF and rbST; Pre, pre-treatment period of rbST; rbST, treatment period of rbST. Significance effect; P<0.05 and trends are declared at 0.05<P<0.10.

Analysis of the possible action of rbST on the concentration ratios among glucose, G6P, and lactose revealed significant decreases (p<0.05) in the G6P/glucose and G6P/lactose ratios, while a relatively constant milk glucose/lactose concentration ratio was apparent throughout lactation (Figure-2b). Comparison of the percent changes in the glucose and G6P concentrations in milk during rbST treatment in all stages of lactation of the MF and NS cows revealed a greater increase and decrease in the concentrations of glucose and G6P, respectively, in milk in the rbST-treated MF cows (Figure-2c). Moreover, the citrate concentration in milk from MF or NS cows was significantly increased after rbST treatment in the early lactation period (p<0.05), while there were no significant changes in the mid- and late lactation periods. The Na^+^, K^+^, and Cl^−^ concentrations and the osmolality in the milk from the MF and NS cows remained unchanged after rbST treatment throughout lactation (Table-2).

## Discussion

Measurement of the environmental temperatures in the NS and MF barns in the afternoon throughout the experimental period showed differences in the AT and RH. Since both NS and MF barns were open types (without walls), both areas received the same reflective re-radiation from the surroundings. Because diurnal variation affects the radiation heat load, especially in the afternoon, it seems that the MF barn exhibited significant alterations of the AT and RH (Figure-1a and b). This implies that the re-radiation heat load at 14.00-15.00 h could be reduced by installing the MF.

Although the MF barn area had a significantly lower mean AT (p<0.05) than the NS barn, this may not be sufficient to eliminate heat stress in cows because the range for THI measured in the daytime in the MF barns throughout the experimental periods remained higher than the THI threshold level (72) for a comfort zone [4]. Indeed, the THI in both NS and MF barns ranged from 80 to 85, similar to the findings in a previous report [18]. Thus, both MF and NS cows would be subjected to moderate heat stress. However, the THI might not accurately reflect the heat stress in the crossbred lactating MF cows, as although the MF cooling system, which delivers a pressurized spray with considerable fanned air movement in the barn, results in higher humidity, it also causes a cooling effect. The MF cows had significantly lower RR and RT than the NS cows, indicating partial alleviation of the heat stress by the MF system, especially in the afternoon [4,18].

The RR and RT were increased during rbST treatment in both MF and NS cows. These results agree with the previous reports on studies of cows treated with rbST associated with a high milk yield [19], and imply that increased heat dissipation would be compromised in rbST-treated cows [20].

In the present study, there were no alterations in the A-V difference of glucose, including the arterial plasma glucose concentrations, among the groups of MF and NS cows with or without rbST treatment. Therefore, the extraction of glucose (A-V difference/arterial concentration) from the blood by the mammary epithelial cells would likely be constant in these circumstances. An increase in glucose uptake by the mammary gland during rbST treatment was not related to the increase in the A-V difference of glucose, but closely related to the increase in MPF. These results suggest that the rate of blood flow to the mammary gland is the determinant of the rate of nutrient uptake by the mammary gland and milk yield. However, several investigations have shown that mammary glucose uptake depends on an increase in plasma glucose concentration during bST treatment [21,22], whereas other studies have found no differences [23]. The present results support the latter observations that there are no changes in the glucose concentration in the arterial plasma in rbST-treated cows.

It is well known that milk yield increases in response to rbST treatment, but the treatment of rbST in crossbred Holstein cattle showed a smaller stimulating effect on milk yield as lactation advanced to the late lactation stage in both MF and NS cows, despite a high MBF level. These results agree with a previous study [2] and indicate that the increased milk yield in response to rbST treatment is not sustained for long and is influenced by the stage of lactation. The previous studies revealed that the increase in MBF in rbST-treated cows coincided with the increased circulating levels of IGF-1 [24]. This implies that rbST plays a role in increasing the MBF indirectly through the action of IGF-1 as a mediator. However, rbST treatment may have an independent direct effect on the mammary gland, since the GH receptor has been reported to localize at the bovine mammary gland [25].

The present results show that rbST treatment increased the concentration of glucose in the aqueous phase of milk, which coincided with increased glucose uptake by the mammary gland in all stages of lactation in both MF and NS cows. The increased milk glucose concentration reflects the increased intracellular glucose concentrations within the mammary epithelial cells [17], since it equilibrates rapidly across the apical membrane of lactating cells [26]. The intracellular glucose freely permeates across the Golgi vesicles and the apical membrane of mammary secretory cells. Mammary epithelial cells cannot synthesize free glucose because of their lack of glucose-6-phosphatase activity [14], so the milk glucose concentration reflects the intracellular glucose concentration, which represents the difference between the rate of glucose transport into the cell and the rate of its intracellular utilization. However, synergistic promotion by exogenous rbST and a low-temperature environment (MF barn) of milk glucose concentration was evident, where the milk glucose concentration was much higher in the MF cows treated with rbST in all stages of lactation. These results indicate mutual potentiation, with a degree of synergism between exogenous rbST and the low-temperature environment, while there was lower synergism with rbST in a high-temperature environment.

In the late stage of lactation, the increase in milk glucose concentration was less evident than at the early and mid-stages of lactation during the rbST treatment in MF and NS cows, although the MBF was still maintained at a high level. Thus, glucose uptake by mammary epithelial cells might be stimulated not only by the direct action of exogenous rbST on MBF but also by other factors. Glucose transporters, mainly GLUT1 and GLUT8, at the mammary cell membrane would be mediators in facilitative glucose uptake [27]. In the late stage of lactation, a decrease in protein content of the mammary tissue, which normally occurs during the declining phase of lactation, may be attributed to the low numbers of glucose transporters in the mammary gland. It is probable that a low number of specific glucose transporters would be apparent in late lactation [27], which would result in the decline in mammary glucose uptake and so contribute to the lower intracellular glucose concentration and reduced milk glucose concentration.

One of the limitations on the uptake rate of glucose in mammary epithelial cells is the activity of the glucokinase enzymes (hexokinases). Low hexokinase activity, and thus a low rate of transformation of glucose into G6P [27], including the low numbers of specific glucose transporters at the mammary cell membrane, may occur in late-stage lactation and so result in a low intracellular glucose concentration in mammary cells and limit the utilization of glucose by the mammary gland. The high MBF during rbST treatment caused a decrease in the glucose transit time, and so the contact time between glucose in the blood and mammary epithelial cells acting as a limiting factor could be ruled out. If this conclusion is correct, it clearly points to the effect of the low number of glucose transporters in late-stage lactation causing a reduction in glucose transport from the plasma into the cell, and implies that the rate of lactose synthesis and low milk yield during late-stage lactation are sensitive to the intracellular glucose concentration. Thus, changes in the availability of intracellular glucose, representing the difference between the rate of glucose transport into the cell and the rate of its intracellular utilization, would be one of the intramammary factors limiting lactose production. This notion warrants further investigation in crossbred dairy cattle in the tropics.

In this study, cows treated with rbST showed an increase in free milk glucose concentration, while the lactose concentration was rather constant in both MF and NS cows, which might reflect the intracellular conditions: Uptake of glucose from the blood circulation, utilization of glucose in the process of lactose synthesis, and the secretion of glucose into the milk. Unlike the milk G6P concentration, a greater reduction in the milk concentration of G6P was obtained with rbST treatment in the MF cows than in the NS cows, which was different from the milk concentration of glucose. This discrimination between the milk glucose and G6P concentrations might occur at the cytosol, instead of the different movement of the two solutes across the apical membrane. Since glucose is a source of lactose synthesis, the concentration of milk glucose may reflect the present vesicular rather than cytosolic concentration. Both glucose and G6P concentrations in milk have been used as potential indicators of the energy status in the mammary gland [28]. A previous study showed an increase in milk G6P concentration and a decrease in milk glucose concentration during periods of energy deficit from starvation [17]. The interrelationship between milk glucose, milk G6P, and lactose concentrations, reflecting the energy metabolism in the cell, likely indicates the energy status of the animal.

The milk concentration of G6P varies much more than those of glucose and lactose. Analysis of the concentration ratios among glucose, G6P, and lactose revealed the possible mechanism of action of rbST that changed the biochemical pathway for lactose synthesis through changes in G6P. Intracellular G6P has been shown to be related to the process of lactose synthesis as a precursor of UDP-galactose, and G6P also serves as an intermediate that can be metabolized through the pathway of glycolysis and the pentose phosphate cycle for the generation of reducing equivalents (NADPH) for the biosynthesis of milk fat. In accordance with this, bST treatment increases the milk fat output and the proportion of long-chain fatty acids in milk fat.

However, rbST treatment in the MF cows reduced the milk G6P concentration, while the milk glucose concentration increased more than in the NS cows treated with rbST and it is possible that the equilibrium between these two metabolites may not be maintained in the cytosol under these conditions. It is known that the uptake of glucose by the transmembrane transport is immediately phosphorylated to G6P by the enzyme hexokinase inside the mammary cells. The G6P pool is fluxed towards for the pentose phosphate pathway, oxidation of G6P to CO2 and lactose synthesis relating to equilibrium between G6P and G1P which UDP-galactose is derived from G1P. The observed decrease in concentrations of G6P in milk during rbST treatment in the MF cows may result from changes in the relevant enzyme activities to establish a new equilibrium. It would be assumed that extracted glucose inside the mammary cells would proceed more for metabolism through phosphorylation by enzyme hexokinase and an apparent decrease in G6P in milk would reflect to an alteration of intracellular G6P pool affecting to its pathway particular equilibrium between G6P and G1P which this process would be further investigated. Hence, the observed increase in the rate of glucose uptake by the mammary gland in MF cows compared with that in NS cows or after rbST treatment may not only be mediated by the level of facilitative glucose transporters but also involve an increased rate of glucose phosphorylation as the hexokinase activity becomes accelerated by the low cytosolic G6P concentrations [26]. Alternatively, the decreased G6P concentrations may reflect an initial decrease in the cytosolic glucose and phosphate concentrations if the regulation of glucose uptake by the mammary gland precedes the increased rate of lactose synthesis and may alter the glucose/G6P ratio inside the cell.

The increased incorporation of glucose into milk during rbST treatment appeared to increase the milk glucose concentration but not the milk citrate level in both MF and NS cows at each stage of lactation. Although a significant change in the glucose uptake was apparent during rbST treatment in both MF and NS cows, an increased level of citrate in milk would be expected, since citrate in milk is known to be derived from blood glucose and acetate. Citrate is thought to enter milk through Golgi vesicles, by the same route as lactose [14]. Lactose and citrate in the Golgi vesicles are secreted into the alveolar lumen by exocytosis [14] because the Golgi and apical membranes of mammary secretory cells are impermeable to lactose but freely permeable to water. Citrate probably exists in the aqueous phase of milk in a variety of chemical forms, such as calcium citrate, citrate^2+^, and citrate^3+^. The relative proportions of each chemical form depend on various factors, such as the concentrations of H^+^, Ca^2+^, and Mg^2+^, so citrate can be considered as a milk buffer.

In this study, the concentrations of milk Na^+^, K^+^, and Cl^−^ did not change during the administration of rbST in both MF and NS cows, although a significant change in the glucose uptake during rbST treatment was apparent in both MF and NS cows. Changes in the concentrations of milk electrolytes would be expected since there is evidence that glucose mobilization inside the cell uses a sodium cotransporter [27]. It has been reported that glucose and electrolytes are secreted across the mammary epithelium from the blood side to the milk side through the membrane route. The mammary gland can generate and maintain steep Na^+^, K^+^, and Cl^−^ gradients between milk and plasma, where the K^+^ concentration in milk, in particular, is considerably higher than that in plasma [4]. Indeed, K^+^ channels have been shown to be localized at the apical membrane of mammary epithelial cells and are associated with milk-borne negative feedback regulation of milk secretion [4]. However, the concentration of these ions in milk did not change during the administration of rbST in both MF and NS cows. This indicates that these ions make a substantial contribution to maintaining the osmolality of milk, and so the present results do not rule out the possibility that exogenous rbST is involved in the maintenance of tissue integrity and function.

## Conclusion

The present study presents evidence that increased availability of intramammary glucose transport is due to both rbST and the mist-fan cooling system. The synergism between the two treatments activated the process of lactose synthesis at different stages of lactation. The synergism between rbST and exposure to a low AT was related to upregulation of the milk glucose concentration and downregulation of the milk G6P concentration. We are pursuing the hypothesis that rbST treatment exerts its galactopoietic action more by altering local intramammary factors than by other extramammary factors. An increase in glucose uptake by the mammary gland in both MF and NS cows during rbST treatment was metabolized through the sufficient pool of G6P, which is a precursor for lactose synthesis and also serves as an intermediate in glycolysis and the pentose phosphate cycle. A marked reduction in milk G6P during the synergism between the rbST treatments of MF cows would probably be due to a larger proportion of the intramammary G6P metabolized through the Embden–Meyerhof pathway compared with that in NS cows.

## Authors’ Contributions

NC, SS, SC, and ST: Contributed to the conception and designed the study. NC and SC: Contributed reagents/materials/analysis tools. NC, SS, SC, and ST: Performed the animal experiments. NC, SS, and SC: Statistical analysis and interpretation. NC and ST: Wrote and revised the paper. All authors read and approved the final manuscript.
